# Anti-malarial efficacy and resistance monitoring of artemether-lumefantrine and dihydroartemisinin-piperaquine shows inadequate efficacy in children in Burkina Faso, 2017–2018

**DOI:** 10.1186/s12936-021-03585-6

**Published:** 2021-01-19

**Authors:** Adama Gansané, Leah F. Moriarty, Didier Ménard, Isidore Yerbanga, Esperance Ouedraogo, Paul Sondo, Rene Kinda, Casimir Tarama, Edwige Soulama, Madou Tapsoba, David Kangoye, Cheick Said Compaore, Ousmane Badolo, Blami Dao, Samuel Tchwenko, Halidou Tinto, Innocent Valea

**Affiliations:** 1 Centre National de Recherche Et de Formation Sur Le Paludisme, Ouagadougou, Burkina Faso; 2 Division of Parasitic Diseases and Malaria, Center for Global Health, Centers for Disease Control and Prevention, US President’s Malaria Initiative, Atlanta, GA USA; 3 Malaria Genetics and Resistance Unit, Department of Parasites and Insect Vectors, Institut Pasteur, Paris, France; 4 IRSS / Unité de Recherche Clinique de Nanoro, Nanoro, Burkina Faso; 5 National Malaria Control Programme, Ouagadougou, Burkina Faso; 6 JHPIEGO/ Improving Malaria Care, Ouagadougou, Burkina Faso

**Keywords:** *Plasmodium falciparum*, Artemether-lumefantrine, Dihydroartemisinin-piperaquine, Efficacy, Burkina faso, Antimalarial

## Abstract

**Background:**

The World Health Organization recommends regularly assessing the efficacy of artemisinin-based combination therapy (ACT), which is a critical tool in the fight against malaria. This study evaluated the efficacy of two artemisinin-based combinations recommended to treat uncomplicated *Plasmodium falciparum* malaria in Burkina Faso in three sites: Niangoloko, Nanoro, and Gourcy.

**Methods:**

This was a two-arm randomized control trial of the efficacy of artemether-lumefantrine (AL) and dihydroartemisinin-piperaquine (DP). Children aged 6–59 months old were monitored for 42 days. The primary outcomes of the study were uncorrected and PCR-corrected efficacies to day 28 for AL and 42 for DP. Molecular markers of resistance to artemisinin derivatives and partner drugs were also analysed.

**Results:**

Of 720 children enrolled, 672 reached study endpoints at day 28, 333 in the AL arm and 339 in the DP arm. PCR-corrected 28-day per protocol efficacy in the AL arm was 74% (64–83%) in Nanoro, 76% (66–83%) in Gourcy, and 92% (84–96%) in Niangoloko. The PCR-corrected 42-day per protocol efficacy in the DP arm was 84% (75–89%) in Gourcy, 89% (81–94%) in Nanoro, and 97% (92–99%) in Niangoloko.

No *Pfk13* mutation previously associated with artemisinin-resistance was observed. No statistically significant association was found between treatment outcome and presence of the 86Y mutation in the *Pfmdr1* gene. There was also no association observed between treatment outcome and *Pfpm2* or *Pfmdr1* copy number variation.

**Conclusion:**

The results of this study indicate evidence of inadequate efficacy of AL at day 28 and DP at day 42 in the same two sites. A change of first-line ACT may be warranted in Burkina Faso.

*Trial Registry* Pan African Clinical Trial Registry Identifier: PACTR201708002499311.

Date of registration: 8/3/2017

https://pactr.samrc.ac.za/Search.aspx

## Background

Despite recent encouraging global progress in malaria control, the brunt of the burden of malaria is still borne by sub-Saharan African countries [1]. In Burkina Faso, malaria is the most frequent cause of hospitalization (57%) and death (36%) in healthcare facilities. *Plasmodium falciparum* is responsible for more than 80% of malaria cases [2, 3]. In 2005, after the decline in efficacy of chloroquine [4–6], artemisinin-based combination therapy (ACT) (artemether-lumefantrine [AL] and artesunate-amodiaquine [ASAQ]) was adopted as the first-line treatment for uncomplicated malaria, but only became available 2 years later [7]. Starting in 2014, the use of ASAQ at public facilities was gradually discontinued as recommended by WHO for areas where seasonal malaria chemoprevention with amodiaquine plus sulfadoxine-pyrimethamine is implemented [8]. Dihydroartemisinin-piperaquine (DP) was added as an additional first-line option in 2017 [9].

The emergence and spread of parasite resistance to artemisinin derivatives and partner drugs used in ACT jeopardizes progress in malaria control [10, 11]. To date, mutations in the propeller domain of the *Pfkelch13* gene have been associated with artemisinin resistance in Southeast Asia and identified in one country in sub-Saharan Africa [12, 13]. Resistance to partner drugs is also a concern; increased copy number of *Plasmodium falciparum multidrug resistance 1* (*Pfmdr1*) and *plasmepsin 2* (*Pfpm2*) genes represent validated molecular signatures associated with resistance to lumefantrine [14] and piperaquine [15, 16], respectively. The World Health Organization (WHO) recommends assessing the therapeutic efficacy and safety of ACT at least every two years [7]. In neighbouring Mali and Niger, recent studies shown high PCR-corrected efficacy for DP (99.4%) and varying efficacy for AL, with 28-day PCR-corrected efficacy measured at 84.5% in a recent published study in Mali [17–19].

In Burkina Faso, studies conducted between 2008 and 2012 have demonstrated PCR-corrected efficacy of AL ranging between 78–91% after 28 days follow-up [20–22], However, these results were generated using alternative statistical methods to those recommended by the WHO (Table 1). This study reports data on the efficacy and the safety of AL and DP in 2017–2018 among children 6–59 months old, in addition to molecular markers associated with anti-malarial resistance, including *Pfkelch13*, *Pfmdr1,* and *Pfpm2* to support evidence-based decisions on malaria treatment policy.

**Table 1:** Results of previous artemether-lumefantrine therapeutic efficacy studies recalculated in accordance with WHO recommendations

				PCR uncorrected 28-day efficacy		PCR corrected 28-day efficacy	
Year of study	Year of publication	Reference	Site	Published^a^	Recalculated^b^	Published^a^	Recalculated^b^
2008–2010	2014	Tinto et al. [22]	Nanoro	46.1	46.1	89.8	81.9
2009	2011	Siribié et al. [20]	Banfora	66.7	66.7	90.5	87.5
2010–2012	2015	Sondo et al. [21]	Nanoro	43.3	43.3	77.8	64.5

^a^Published results

^b^Recalculated using WHO per protocol definition [7]

## Methods

### Study sites

**Fig. 1:**
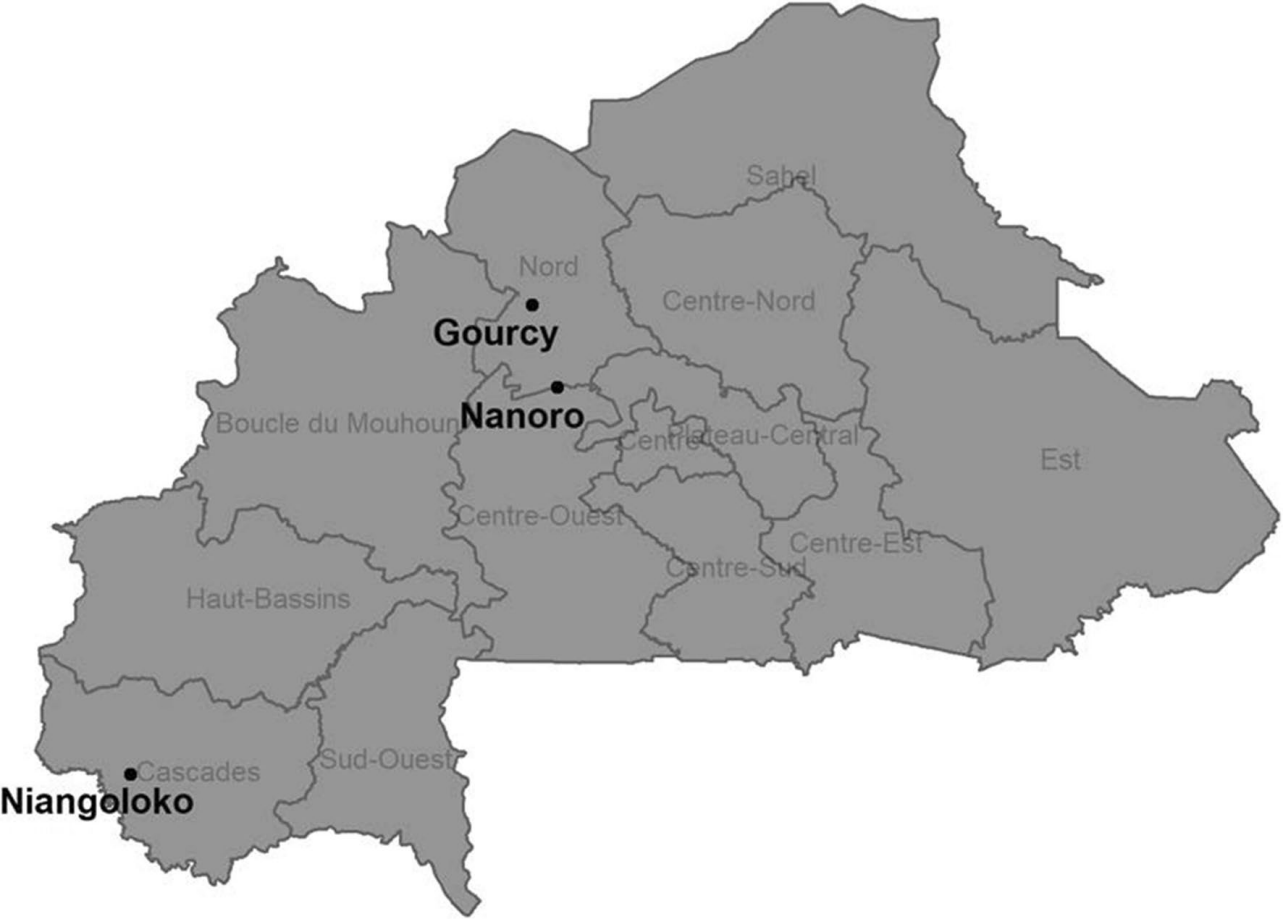
Map of therapeutic efficacy study sites, Burkina Faso, 2017–2018

The study was conducted in three sites representing differing malaria transmission zones in Burkina Faso:

- Niangoloko: The Niangoloko Medical Centre located in the Banfora Health District; malaria transmission occurs year-round with a peak during the rainy season (May–October). Malaria incidence is reported as 2349 per 1000 among children under 5 years old according to routine surveillance data [23].
- Nanoro: The Nanoro and Temnaoré primary health facilities in the Nanoro Health District; malaria transmission is stable, seasonal and occurs throughout the rainy season (May–October). Malaria incidence is reported as1979 per 1000 among children under 5 years old according to routine surveillance data [23].
- Gourcy: The Gourcy District Hospital located in the Gourcy Health District; malaria transmission is stable, seasonal and occurs throughout the rainy season (June–September); malaria incidence is reported as 1526 per 1000 in children under 5 years old according to routine surveillance data [23] (Fig. 1).

### Study design and randomization

This was a phase IV randomized, open label, multi-site study assessing the efficacy and safety of AL and DP based on the WHO protocol for the surveillance of anti-malarial drug efficacy [7]. Children with uncomplicated *P. falciparum* malaria who met the study inclusion criteria were screened, enrolled, assigned to treatment with AL or DP by block randomization and monitored for 42 days.

### Sample size estimation

Considering a maximum acceptable treatment failure rate of 10% with a confidence level of 90% and precision of 5%, the sample size needed was estimated at 105 patients per arm in each site; enrolment of 120 participants per arm was planned to account for withdrawal or loss to follow-up. According to the WHO, in order for the study to be representative, a minimum sample of 50 patients is required, regardless of the rates of failure.

### Screening and recruitment

Children aged between 6–59 months with fever or history of fever in the previous 24 h seeking treatment at the targeted health facilities were examined by nurses and tested for malaria with a rapid diagnostic test (RDT) (Malaria Ag Pf/Pan, SD Bioline). Children with a positive RDT and no other illness requiring immediate attention were referred to the study team for screening procedures including clinical examination and malaria microscopy. Children were enrolled in the study if the following criteria were met: (a) mono-infection with *P. falciparum* detected by microscopy, (b) asexual parasite count of 2000–200 000/μl, (c) axillary temperature ≥ 37.5 °C or reported fever during the previous 24 h, (d) haemoglobin level ≥ 5 g/dl, (e) ability to swallow oral medication, (f) ability and willingness of caregivers to comply with the protocol for the duration of the study, (g) no history of effective anti-malarial treatment in the preceding 72 h and (h) written informed consent of a parent or guardian. Other selection criteria were evaluated according to the WHO standardized protocol [7]. Children who did not meet the screening criteria received free medication according to national guidelines.

### Examination of malaria parasites by microscopy and haemoglobin level measurement

Capillary blood was collected by finger prick and used to prepare thick and thin blood smears in duplicate during study visits. Slides were independently read by two qualified microscopists; results were averaged if they were consistent (< 20% for the parasite density estimation). If results from the first two readers were discordant, the slide was read by a third qualified microscopist. The final parasitaemia was calculated as the average of the two closest parasitaemia estimations. Only asexual forms were included in the parasite density estimation.

Haemoglobin was measured in capillary blood using a portable haemoglobin analyzer HemoCue Hb 201 System (HemoCue AB, Angelholm, Sweden) on days 0, 14 and 28 or any other days on request of clinicians.

A post-hoc quality control was done on a subset of slides (n = 233) at the Noguchi Memorial Institute for Medical Research whereby two microscopists blinded to the original results read the slides. An additional 10% of the subset were read by a third and fourth microscopist.

### Treatment and clinical monitoring during follow-up

Dispersible 20/120 mg AL tablets (Coartem^®^) were purchased from LABOREX, a national drug supplier, and DP 20/160 mg and 40/320 mg tablets (D-Artepp^®^) were provided by Guilin Pharmaceutical in China. At each study site, a nurse managed the study drugs per the manufacturer’s instructions and with recording daily temperature in limited access and air-conditioned storage room. The certificates of analysis of each batch of drugs used on site during the study were requested and archived in the investigator site file.

Each child received a 3-day course of the assigned study drug according to the national guidelines for the treatment of uncomplicated malaria. AL was given in tablets containing 20 mg of artemether and 120 mg of lumefantrine and was given in two daily doses. Children weighing 5–9 kg were given one half of a tablet; children weighing 10–14 kg were given one tablet, and children weighing 15–24 kg were given two tablets. DP was given once per day. Children weighing 5–8 kg and 8–11 kg were given tablets containing 20 mg of dihydroartemisinin and 160 mg of piperaquine at 1 and 1.5 tablets, respectively. Children weighing 11–17 kg and 17–25 kg were given tablets containing 40 mg of dihydroartemisinin and 320 mg of piperaquine at 1 and 1.5 tablets, respectively. The three once-daily doses for DP and the three twice-daily doses for AL doses were directly supervised by the study team. Doses were not systematically given with food. If a participant vomited within 30 min following drug administration, the full dose was re-administered. In case of persistent vomiting, the child was excluded and treated according to national guidelines for the treatment of severe malaria.

Children were followed for 42 days regardless of drug arm with clinical and laboratory examinations performed on days 0, 1, 2, 3, 7, 14, 21, 28, 35 and 42. Caregivers of study participants were asked to bring their children to the health facility at any time if they felt unwell between scheduled visits. Children who did not attend scheduled visits by mid-day were visited at home by a member of the study team and their caregivers encouraged to bring the child to the health facility for the study procedures. Children were classified as lost to follow-up if they were not seen for each scheduled visit through the end of the study. Children presenting with recurrent parasitaemia were treated per the national guidelines based on clinical presentation [9]. Parents/guardians of participants were provided transportation costs at each scheduled visit.

Drug safety was monitored through questionnaires administered to caregivers and during clinical examination. A severity grading scale, based on the WHO toxicity grading scale was used to assess the severity of reported adverse events and clinical examination findings [24]. Patients with adverse events were assessed, managed in accordance with their clinical status and followed until resolution or stabilization.

All participants were assigned one of the following treatment outcomes according to the WHO guidelines [7]:

- Early treatment failure: danger signs or severe malaria on days 1–3 with parasitaemia, higher parasitaemia on day 2 than day 0, parasitaemia on day 3 with a fever or day 3 parasitaemia ≥ 25% than day 0,
- Late clinical failure: danger signs or severe malaria with parasitaemia or parasitaemia with fever between days 4 and last day of follow-up,
- Late parasitological failure: parasitaemia between day 7 and last day of follow-up in absence of a fever,
- Adequate clinical and parasitological response (ACPR): absence of parasitaemia on last day of follow-up, irrespective of fever status, or
- Removed or lost to follow-up.

### Sample processing and molecular analysis

Dried blood spots (DBS) were prepared from 2 to 3 drops of capillary blood spotted on Whatman 903 filter paper on day 0 and any day of recurrence of parasitaemia after day 7. Parasite DNA was extracted using QIAamp DNA blood mini kits (Qiagen, Valencia, CA USA) according to the manufacturer’s instructions. *Plasmodium* species was confirmed by PCR on day 0 samples for all treatment failures and a subset of samples for participants classified as ACPR [25]. For recurrent parasitaemias, genotyping using amplification of the *merozoite surface proteins 1* and *2* (*msp1* and *msp2*), and *glutamine-rich protein* (*glurp*) markers was performed on paired samples obtained from participants on day 0 and day of failure.

Primers designed to amplify three allelic families from block two of *msp1* (K1, MAD20, R033), two allelic families from *msp2* (FC27 and IC/3D7), and the polymorphic region of *glurp* were used in PCR amplification and analysis as previously described [26, 27]. Products from paired samples were loaded adjacent to each other. Gels were stained with ethidium bromide and visualized under UV illumination. Band sizes of PCR products across the three markers were measured visually and compared for paired day 0 and day of failure samples. Band sizes that were equal on day 0 and day of failure were considered to match. If there was at least one matching band in any allelic family for all three markers, the failure was classified as a recrudescence (regardless of whether there were additional or missing alleles). If there were no shared alleles for at least one marker, the failure was classified as a reinfection. If the amplification products failed to result in sharp, defined bands in both day 0 and day of failure samples for a marker, that marker was not used for reinfection and recrudescence determination, but the aforementioned classification criteria were applied for the markers that were amplified.

### Markers of anti-malarial drug resistance

All treatment failures and a random subset of samples from patients classified as ACPR were analysed for markers of anti-malarial drug resistance to explore associations between treatment outcome and presence of mutations. DNA extracts from day 0 were analysed to detect the presence of mutations in the propeller domain of *Pfkelch13* (PF3D7_1343700) and the presence of the N86 allele as previously described and compared across participants classified as ACPR, reinfection, and recrudescence [28, 14]. Sequencing reactions were carried out with a CFX96 Touch BioRad (Marnes-la-Coquette, France). Amplicons were sent to Eurofins Germany for sequencing, and DNA sequences were analysed to identify specific single nucleotide polymorphisms (SNPs) related to anti-malarial resistance. Electropherograms were analysed with CEQ2000 genetic analysis software (Beckman Coulter, Villepinte, France). Parasites with mixed alleles (in which both wild-type and mutant alleles were present) were included in counts for both wild-type and mutant alleles. *Plasmepsin-2 (Pfpm2,* PF3D7_1408000) and *Pfmdr1* (PF3D7_0523000) copy number variation (CNV) were measured by qPCR using a CFX96 Touch (Bio-Rad, France), relative to the single copy of the β-tubulin gene (used as reference gene), as previously described [16]. Molecular analyses were done at the Malaria Genetics and Resistance Unit, Department of Parasites and Insect Vectors, Institut Pasteur in Paris, France.

### Statistical analysis

Data collected on case report forms were double entered in CSPro 7.0 (US Census Bureau, Washington DC, USA) then imported and checked in Excel (Microsoft, Redmond, USA). Data were analysed with the WHO Excel software template [29], MedCalc version 12 (Mariakerke, Belgium) and R (R Foundation for Statistical Computing, Vienna, Austria). Patients with new infections during the follow-up period and patients with indeterminate PCR genotyping data were excluded from the PCR-corrected per-protocol analysis and censored on day of failure in the Kaplan–Meier estimates. Results were calculated to day 28 for the AL arms and to days 28 and 42 for the DP arms due to the longer half-life of piperaquine. Presence of *Pfkelch13* and *Pfmdr1* mutations were compared between day 0 samples from those who successfully cleared their initial infection (outcome of ACPR or reinfection) versus day 0 samples from those who developed recrudescent infection using Fisher’s exact test. These groups were also used to compare CNV at different cutoffs (1.5, 2, 2.5, and 3) and as a continuous variable. *Pfpm2* CNV was analysed in the DP arm and *Pfmdr* CNV was analysed in the AL arm.

### Ethical considerations

The study protocol was approved by the Institutional Ethics Committee of CNRFP and the Health Research Ethics Committee of Burkina Faso and was conducted in accordance with International Conference on Harmonization and Good Clinical Practices. The trial was registered in the Pan African Clinical Trial Registry (PACTR identifier: PACTR201708002499311) and approved by the Regulatory authority from the Ministry of Health. Staff from the Centers for Disease Control and Prevention (CDC) provided technical assistance; the protocol was approved as a non-research program evaluation by the Office of the Associate Director for Science, Center for Global Health at CDC.

## Results

### Study profile

**Fig. 2:**
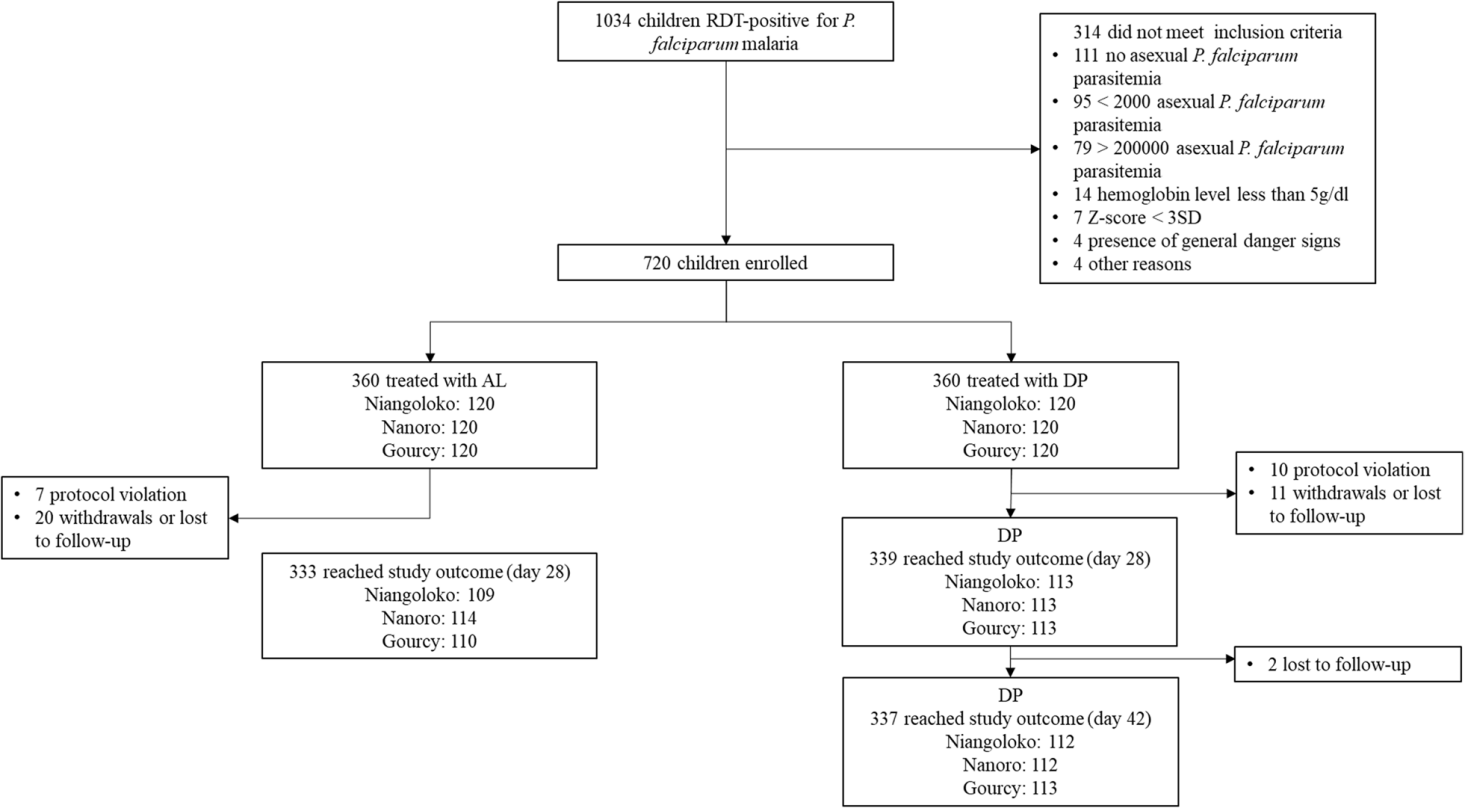
Therapeutic efficacy study flow chart, Burkina Faso, 2017–2018. RDT: rapid diagnostic test. AL: artemether-lumefantrine. DP: dihydroartemisinin-piperaquine. Danger signs include inability to drink or breastfeed, repeated vomiting (> 2 times in 24 h), convulsions, unconscious state, inability to sit or stand

Recruitment took place between November 2017 and September 2018. At day 28 of follow-up, 333 and 339 children in the AL and DP arms, respectively reached study endpoints. At day 42 of follow-up, 337 children were evaluated in the DP arm (Table 2, Fig. 2). All groups received the recommended dose of AL or DP except that the dose of AL given to children weighing 5–9 kg was a half tablet instead of 1 full tablet due to an error in the study protocol approved by ethics committees and regulatory authority.

**Table 2:** Baseline characteristics of participants enrolled in the 2017–2018 therapeutic efficacy study, Burkina Faso

	Niangoloko		Nanoro		Gourcy	
	AL	DP	AL	DP	AL	DP
Screened	375		335		324	
Included in analysis (%)	116	118	117	113	120	118
Age in months. mean (SD)	33.6 (13.2)	33.6 (13.2)	33.6 (13.2)	31.2 (12.0)	34.8 (12.0)	34.8 (12.0)
Sex (male) n (%)	58 (50)	68 (57.6)	57 (48.7)	61 (53.0)	59 (49.2)	62 (52.5)
Weight (kg). mean (SD)	12.0 (2.7)	12.1 (2.7)	11.6 (2.0)	11.2 (2.2)	11.8 (2.2)	11.7 (2.3)
Temperature in °C, mean (SD)	38.3 (1.0)	38.3 (1.0)	38.2 (1.1)	38.1 (1.1)	38.1 (1.2)	38.2 (1.0)
Parasitaemia day 0^a^	36,594	36,571	48,891	42,393	42,116	33,536
Hemoglobin in g/dl. mean (SD)	10.2 (1.7)	10.0 (2.9)	9.9 (1.7)	10.1 (1.6)	10.0 (1.5)	9.9 (1.6)

*AL* artemether lumefantrine, *DP* dihydroartemisinin piperaquine, *SD* standard deviation

^a^geometric mean parasite density (asexual parasites/µl)

### Clinical and parasitological response

The 672 children reaching endpoints were considered in the efficacy estimates. At day 3, post-treatment parasitaemia was detected in 6 subjects (2%) in the DP arm and 1 (0.2%) in the AL arm. In the AL arm, there were 177 late treatment failures, of which 54 (31%) were classified as recrudescences, 107 (60%) as reinfections, and 16 (9%) were non-amplified or not available. The uncorrected 28-day AL per-protocol efficacy was 65% (55–74%) in Niangoloko, 27% (95% confidence intervals (CI) 19–36%) in Nanoro, and 48% (95% CI 39–58%) in Gourcy. The PCR-corrected Kaplan–Meier efficacy was 92% (95% CI 84–96%) in Niangoloko 74% (95% CI 64–83%) in Nanoro, and 76% (95% CI 66–83%) in Gourcy. Excluding the underdosed 5–9 kg weight group, the PCR uncorrected 28-day AL per-protocol efficacy was 68% (95% CI 57–77%) in Niangoloko, 28% (95% CI 20–38%) in Nanoro, and 44% (95% CI 33–55%) in Gourcy. The PCR-corrected Kaplan–Meier efficacy was 92% (95% CI 83–96%) in Niangoloko, 77% (95% CI 65–85%) in Nanoro, and 73% (95% CI 61–81%) in Gourcy.

In the DP arm, there were 82 late treatment failures at day 42, 26 (32%) of which were classified as recrudescences, 48 (59%) as reinfections, and 8 (10%) were non-amplified or not available. The uncorrected 42-day DP per-protocol efficacy was 88% (95% CI 80–94%) in Niangoloko, 64% (95% CI 55–73%) in Nanoro, and 69% (95% CI 60–77%) in Gourcy. The PCR-corrected 42-day Kaplan–Meier efficacy for DP was 97% (95% CI 92–99%) in Niangoloko, 89 (95% CI 81–94%) in Nanoro, and 84% (95% CI 75–89%) in Gourcy (Table 3). Based on genotyping of day 0 samples from late treatment failures, the proportion of infections with a multiplicity of infection > 1 was estimated at 83% (238/286). The risk of positivity on day 3 was statistically associated with a parasite density > 50,000 parasites/µL at day 0 (*p* = 0.02, Chi-squared test). Raw genotyping data are available in the Additional file 1.

**Table 3:** Uncorrected and PCR-corrected efficacy estimates, Burkina Faso therapeutic efficacy study, 2017–2018

	Niangoloko			Nanoro			Gourcy		
	AL	DP	DP	AL	DP	DP	AL	DP	DP
	28 Days	28 Days	42 Days	28 Days	28 Days	42 Days	28 Days	28 Days	42 Days
Enrolled	120	120	120	120	120	120	120	120	120
Reached study outcome (%)	109 (90.8)	113 (94.2)	112 (93.3)	114 (95.0)	113 (94.2)	112 (93.3)	110 (91.7)	113 (94.2)	113 (94.2)
Day 3 parasitaemia (%)	2 (2.0)	0 (0.0)	0 (0.0)	1 (0.9)	2 (1.8)	2 (1.8)	0 (0.0)	4 (3.5)	0 (0.0)
Early treatment failure (%)	0 (0.0)	0 (0.0)	0 (0.0)	1 (0.9)	2 (1.8)	2 (1.8)	0 (0.0)	4 (3.5)	4 (3.5)
Late clinical failure (%)	13 (11.9)	2 (1.7)	4 (3.6)	35 (30.7)	2 (1.8)	16 (14.3)	25 (22.7)	2 (1.8)	11 (9.7)
Late parasitological failure (%)	25 (22.9)	1 (0.9)	9 (8.0)	47 (41.2)	6 (5.3)	22 (19.6)	32 (29.1)	3 (2.7)	20 (17.7)
Recrudescence	8	1	3	22	3	9	24	3	14
Reinfection	23	2	7	55	6	24	29	2	17
No PCR result available	7	0	3	5	1	5	4	0	0
ACPR	71	111	99	31	103	72	53	104	78
% ACPR Uncorrected (95% CI)	65.1 (55.4–74.0)	98.2 (93.8–99.8)	88,4 (80.0–93.7)	27.2 (19.3–36.3)	91.2 (84.3–95.7)	64.3% (54.7–73.1)	48.2 (38.6–57.9)	92.0 (85.4–96.3)	69.0 (59.6–77.4)
% ACPR PCR-Corrected (95% CI)	89.9 (81.0–95.5)	99.1 (95.1–100)	97.1 (91.6, 99.4	57.4 (43.2–70.8)	97.2 (92.0–99.4)	86.7 (77.5–93.2	68.8 (57.3–78.9)	93.7 (87.4.-97.4)	81.3 (72.0–88.5)
Kaplan–Meier cumulative Efficacy									
Uncorrected (95% CI)	65.5 (55.8–73.6)	98.2 (93.1–99.6)	88.5 (88.1–93.2)	27.8 (19.9–36.2)	91.2 (84.2–95.1)	64.3 (54.7–72.4)	48.7 (39.1–57.6)	92.0 (85.1–95.7)	69.1 (59.7–76.7)
PCR-corrected (95% CI)	91.6 (83.9–95.7)	99.1 (93.9–99.9)	97.2 (91.7–99.1)	74.4 (63.5–82.5)	97.3 (92.0–99.1)	88.7 (80.5, 93.6)	75.6 (65.8–83.0)	93.9 (87.6–97.0)	83.6 (75.2–89.3)

*AL* artemether lumefantrine, *DP* dihydroartemisinin piperaquine, *CI* confidence intervals, *PCR* polymerase chain reaction, *ACPR* adequate clinical and parasitological response

### Safety

The most frequently reported adverse events during 28 days of follow-up were vomiting (12.4%), cough (5.4%) and abdominal pain (2.4%) (Table 4). No meaningful variation in haemoglobin level was observed in any study arm (Additional file 2: Table S1). One serious adverse event occurred. A 35-month old girl in the Gourcy AL arm died on day 37. She was seen on day 36 for report of fever which had been treated at home with 250 mg of paracetamol on the previous day. A blood smear examination found 15,346 asexual *P. falciparum* parasites/µl. She was immediately treated with oral quinine as per the national treatment guidelines [9]. The caregivers were advised to bring the child back if her condition did not improve. That night, as the condition of the child was deteriorating, she was taken to a traditional healer from whom she received an unknown treatment. The child died at home later the same day.

**Table 4:** Adverse events reported among participants enrolled in 2017–2018 therapeutic efficacy study, Burkina Faso

	Niangoloko		Nanoro		Gourcy	
	AL (n = 116)	DP (n = 118)	AL (n = 117)	DP (n = 114)	AL (n = 120)	DP (n = 118)
Itchiness	0	0	0	0	1	0
Otitis media	1	0	0	0	0	0
Cough	14	14	4	1	3	2
Abdominal pain	2	7	2	5	0	1
Skin rash	0	2	2	0	0	1
Oral thrush	0	0	0	0	0	0
Furunculosis	0	1	0	0	0	0
Vomiting	12	23	15	18	6	13
Death	0	0	0	0	1	0

*AL* artemether lumefantrine, *DP* dihydroartemisinin piperaquine

### Assessment of molecular markers associated with antimalarial drug resistance

A total of 373 day 0 samples were tested for the presence of SNPs; results were interpretable for 367 (98%). Most parasites (95.9%) had a wild-type *Pfkelch13* gene. Eight *P. falciparum* isolates (2.3%) had synonymous mutations and 7 (1.9%) had non-synonymous mutations. None of the non-synonymous mutations found have been associated with artemisinin resistance [12]. Of note, *Pfkelch13* N629Y (n = 1) and V517I (n = 2) mutants were observed in day 0 isolates collected from patients treated with AL and classified as recrudescences (Table 5). No associations between day-3 parasitaemia or treatment outcome and presence of *Pfkelch13* mutation was found (*p* = 1, *p* = 0.14, Fisher’s exact test).

**Table 5:** Prevalence of *Pfk13, Pfmdr1,* and *Plasmepsin-2* polymorphisms in day 0 samples (subset of day 0 ACPR and all day 0 reinfection samples) and day 0 samples for recrudescences, Burkina Faso therapeutic efficacy study, 2017–2018

Polymorphism	All day 0 samples			Reinfection (day 0) + ACPR			Recrudescent (day 0)^a^			*p*^b^
	n	Total Samples	Percent (%)	n	Total samples	Percent (%)	n	Total samples	Percent (%)	
*Pfk13*										
Samples successfully sequenced	367	373	98.4	265	270	98.1	102	103	99.0	**–**
Wild type (no mutations detected)	352	367	95.9	257	265	97.0	95	102	93.1	Ref
Mutant^c^	15	367	4.1	8	265	3.0	7	102	6.9	0.14
*Pfmdr1 codon* 86										
Samples successfully sequenced	318	362	87.8	228	251	90.8	90	94	95.7	–
N86	303	318	95.3	215	228	94.3	88	90	97.8	Ref
86Y	10	318	3.1	8	228	3.5	2	90	2.2	0.73
N/Y (mixed infection)	5	318	1.6	5	228	2.2	0	90	0.0	0.33
*Pfpm2* copy number (DP arm only)										
Samples successfully sequenced	131	136	96.3	105	109	96.3	26	27	96.3	–
< 1.5	93	131	71.0	74	105	70.5	19	26	73.1	1.00
≥ 1.5	38	131	29.0	31	105	29.5	7	26	26.9	
< 2	106	131	80.9	85	105	81.0	21	26	80.8	1.00
≥ 2	25	131	19.1	20	105	19.0	5	26	19.2	
< 2.5	110	131	84.0	88	105	83.8	22	26	84.6	1.00
≥ 2.5	21	131	16.0	17	105	16.2	4	26	15.4	
< 3	116	131	88.5	94	105	89.5	22	26	84.6	0.50
≥ 3	15	131	11.5	11	105	10.5	4	26	15.4	
Geometric Mean (SD)	1.39 (1.66)			1.40 (1.66)			1.34 (1.72)			0.78
*Pfmdr1* copy number (AL arm only)										
Samples successfully sequenced	235	237	99.2	159	161	98.8	76	76	100	
< 1.5	183	235	77.9	125	159	78.6	58	76	76	0.74
≥ 1.5	52	235	22.1	34	159	21.4	18	76	24	
< 2	205	235	87.2	139	159	87.4	66	76	87	1
≥ 2	30	235	12.8	20	159	12.6	10	76	13	
< 2.5	213	235	90.6	143	159	89.9	70	76	92	0.76
≥ 2.5	22	235	9.4	16	159	10.1	6	76	8	
< 3	225	235	95.7	151	159	95.0	74	76	97	0.51
≥ 3	10	235	4.3	8	159	5.0	2	76	3	
Geometric mean	1.21 (1.54)			1.20 (1.55)			1.24 (1.51)			0.38

^a^Includes recrudescent samples from 42 days of follow up for both arms

^b^Fisher's exact test for proportions; independent t-test for means; comparison between reinfection plus adequate clinical and parasitological response (ACPR) versus recrudescent samples

^c^Mutations include: C469C, E509E, E567E, G496G, V589V, R561R, N629Y, A578S, V517I, and V589A

To investigate mutations in the *Pfmdr1* gene, 362 samples were tested; 318 (88%) yielded interpretable data. Most isolates (303/318; 95.3%) had the N86 allele; 15/318 (4.7%) carried the 86Y mutation only; 5 samples had mixed infections consisting of parasites with a wild-type allele and 86Y mutation. No statistically significant association was found between treatment outcome and presence of the 86Y mutation (Table 5).

**Fig. 3:**
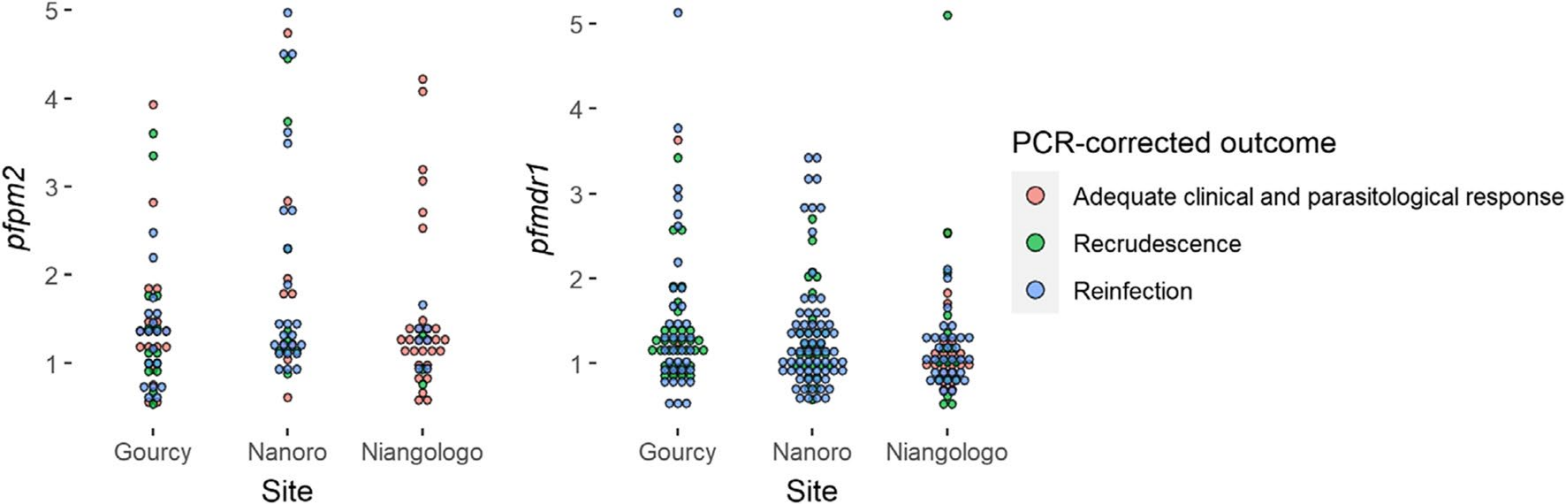
Copy number variation in dihydroartemisinin-piperaquine arm for *pfpm2* copy number (n = 131) and artemether-lumefantrine arm for *pfmdr1* (n = 235) in pre-treatment samples collected for therapeutic efficacy monitoring in three sites in Burkina Faso

In the DP arm, 131 of 136 (96.3%) samples were amplified for measurement of *Pfpm2* CNV. There was no statistically significant association between treatment failure and *Pfpm2* CNV whether using discrete cut-off points or continuous values. In the AL arm, 235 of 237 (99.2%) samples were amplified for analysis of *Pfmdr1* copy number. No statistical evidence of association between treatment failure and CNV was found for *Pfmdr1* (Table 5, Fig. 3).

In the microscopy quality control analysis, the random sample of slides read were concordant, giving a concordance of 88%.

## Discussion

The present study was designed to assess the therapeutic efficacy of AL and DP, two artemisinin-based combinations (ACT) recommended for the treatment of uncomplicated malaria by the NMCP in Burkina Faso among children 6–59 months old. Molecular markers associated with anti-malarial resistance were investigated to provide insight into efficacy results.

In this study, the PCR-corrected ACPR rates at day 28 and 42 were found to be above or equal to 90% in all the sites for DP at day 28, but below 90% for Nanoro and Gourcy at day 42, with confidence intervals spanning above 90% in Gourcy. In the AL arm the 28-day PCR-corrected ACPR rates were below 80% in two of the three sites (Nanoro and Gourcy), indicating inadequate efficacy of this ACT. The estimated proportion of treatment failure with AL (> 10%) contrasts with the low proportion of children still parasitemic at day 3 (< 2%) and the absence of *Pfkelch13* mutants associated with artemisinin resistance in Day 0 *P. falciparum* isolates, suggesting that these *P. falciparum* parasite populations are susceptible to artemisinin derivatives but may have decreased susceptibility to lumefantrine. However, no difference in mutations in the *Pfmdr1* N86 allele was found between samples from those who cleared their initial infection versus those who would experience a recrudescent infection. However, there were few participant samples found to carry the 86Y allele, so there may not have been power to detect a meaningful difference. Furthermore, examining more markers on the *Pfmdr1* gene may provide more information about the association between presence of mutations and treatment outcome. The day 3 positivity rates observed in both treatment arms were low (0–2%), providing evidence of effective and rapid impact of artemisinin derivatives in reducing the parasite biomass [30].

The PCR-uncorrected treatment failure rates on day 28 observed in the AL arm (ranging from 27 to 65%) demonstrate a lack of post-treatment prophylactic effect in a setting of high endemicity. This scenario, in which one-third to nearly three-quarters of children return with recurrent parasitaemia after taking AL, has important implications for the health of individuals [31–33], the functioning of the healthcare system, and the need for repeated malaria treatment.

Proportions of treatment failures from previous therapeutic efficacy studies completed in Burkina Faso since 2005 vary from 9.5% to 23.8% [20–22]. In these studies, reinfections, ascertained by PCR genotyping, were classified as ACPR (i.e., treatment successes) whereas, according to the WHO guidelines, reinfections should be excluded in the per protocol analysis and censored on the day of reinfection in the Kaplan–Meier analysis [7]. Applying the WHO guidelines to data from these studies, there is evidence that treatment failure rates have been above 10% for AL in Burkina Faso since 2009, which is more consistent with the current study and shows that the efficacy of AL has been inadequate for several years (Table 1). Continued monitoring in sites that represent the different epidemiologic zones is important to examine heterogeneity of the failure rate by geographic area.

All non-serious adverse events were resolved by the end of the follow-up. Only one serious adverse event occurred in the AL group. The cause of the death was not confirmed but was unlikely associated with the study drug or concomitant medication.

Of six artemisinin-based combinations recommended by the WHO, four are used as first-line treatment of uncomplicated *P. falciparum* malaria in Africa: AL, ASAQ, DP, and artesunate-pyronaridine (AS-PYR) [34]. However, ASAQ is no longer recommended for use in Burkina Faso because SMC with SP-AQ is used nationwide [8], and the present study shows evidence of inadequate 28-day efficacy for AL and inadequate 42-day efficacy for DP. The last of the four, AS-PYR, has revealed promising results in initial clinical trials in Burkina Faso [35] and warrants further examination in future efficacy studies in the country. However, introducing a new ACT has cost implications for procurement, training of staff, and safety monitoring.

## Limitations

There are several limitations to this study. First, the dose of AL given in the 5–9 kg weight group, was a half tablet instead of 1 tablet due to an error in the drugs dosing table in the protocol approved by the ethics committees and regulatory authority. However, removing this group from the analysis did not change the efficacy results significantly. Additionally, a fatty food was not given systematically to the children in the AL group as recommended by the manufacturer. Some studies have shown that those taking AL on an empty stomach absorb less than 10% of the lumefantrine in the dose given [36, 37]. The absence of pharmacokinetic data on lumefantrine did now allow for inference of adequate dosing or absorption of AL. Adding this to future studies may provide insight into associations between AL absorption and drug efficacy.

Many samples from late treatment failures failed to amplify and were thus excluded in the PCR-corrected per protocol analysis and censored on the day of recurrence in the Kaplan–Meier estimates [7]. This classification method may lead to biased and imprecise efficacy estimates because indeterminate samples are classified the same way as reinfections in the calculation of PCR-corrected efficacy [38]. Among those that did amplify for *msp1* and *msp2*, many failed to amplify for *glurp*, leaving only two markers for determining whether these failures were recrudescences or reinfections. More precise recommended genotyping and analysis methods would help to ameliorate this limitation in future anti-malarial efficacy studies.

Finally, there are limitations in interpretation of *Pfpm2* and *Pfmdr1* CNV in settings where there is high multiplicity of infection due to the inability to quantify the proportion of clones harbouring single or multicopies of *Pfpm2* and *Pfmdr1*.

## Conclusion and recommendations

The results of this study indicate inadequate efficacy of AL in two sites representing areas with shorter malaria transmission seasons in Burkina Faso, consistent with re-analysis of previous efficacy studies. There is evidence of inadequate efficacy of DP at day 42 in the same two sites. Consideration of a change of first-line ACT may be warranted in Burkina Faso. Further in vivo efficacy studies are planned, which will include dosing of AL according to the manufacturer’s instructions, day 7 blood anti-malarial concentration, increased sample size to account for the assumption of a higher failure rate and high rate of reinfection, and inclusion of artesunate-pyronaridine.

## Supplementary Information

**Additional file 1.** Raw genetic data from day 0 and day of failure samples used to distinuish reinfections from recrudescences among late treatment failures, therapeutic efficacy monitoring, Burkina Faso, 2017–2018.

**Additional file 2: Table S1.** Hemoglobin g/dl measured among participants in therapeutic efficacy monitoring, 2017-2018, Burkina Faso

## Data Availability

All data generated or analysed during this study are available from the corresponding author upon reasonable request.
