# Supplementary material for: Anti-malarial efficacy and resistance monitoring of artemether-lumefantrine and dihydroartemisinin-piperaquine shows inadequate efficacy in children in Burkina Faso, 2017–2018
Source: Malar J. 2021 Jan 19;20:48. doi: 10.1186/s12936-021-03585-6 (PMC7816451; doi:10.1186/s12936-021-03585-6)
Supplement: Supplementary file 2 — Additional file 2: Table S1. Hemoglobin g/dl measured among participants in therapeutic efficacy monitoring, 2017-2018, Burkina Faso [file 12936_2021_3585_MOESM2_ESM.docx]

Additional file 2 table S1: Hemoglobin g/dl measured among participants in therapeutic efficacy monitoring, 2017-2018, Burkina Faso

|  | Hemoglobin in g/dl, mean (SD) at day 0 | Hemoglobin in g/dl, mean (SD) at day 14 | Hemoglobin in g/dl, mean (SD) at day 28 |
| --- | --- | --- | --- |
|  | **Niangoloko** | | |
| **AL** | 10.2 (1.7) | 10.0 (1.3) | 10.5 (1.3) |
| **DP** | 10.0 (2.92) | 9.8 (1.2) | 10.6 (1.6) |
|  | **Nanoro** | | |
| **AL** | 9.9 (1.6) | 10.2 (1.2) | 10.2 (1.5) |
| **DP** | 10.1 (1.6) | 10.1 (1.1) | 10.7 (1.2) |
|  | **Gourcy** | | |
| **AL** | 10.0 (1.5) | 9.8 (1.2) | 9.7 (1.5) |
| **DP** | 9.9 (1.6) | 9.7 (1.3) | 10.3 (1.4) |

AL: Artemether Lumefantrine

DP: Dihydroartemisinin Piperaquine

SD: Standard deviation
